# EEG repetition and change detection responses in infancy predict adaptive functioning in preschool age: a longitudinal study

**DOI:** 10.1038/s41598-023-34669-9

**Published:** 2023-06-20

**Authors:** Florence Deguire, Gabriela López-Arango, Inga Sophia Knoth, Valérie Côté, Kristian Agbogba, Sarah Lippé

**Affiliations:** 1 Psychology Department, University of Montreal, Marie Victorin Building, 90 Vincent-D’Indy Avenue, Montreal, QC Canada; 2 Pôle en neuropsychologie et neuroscience cognitive et computationnelle (CerebrUM), University of Montreal, Marie Victorin Building, 90 Vincent-D’Indy Avenue, Montreal, QC Canada; 3 Research Center of the CHU Sainte-Justine, University of Montreal, 3175 Chemin de la Côte-Sainte-Catherine, Montreal, QC Canada; 4 École de technologie supérieure, University of Quebec, 1100 Notre-Dame W, Montreal, QC Canada

**Keywords:** Neuroscience, Cognitive neuroscience, Learning and memory, Sensory processing

## Abstract

Neurodevelopmental disorders (NDDs) are mostly diagnosed around the age of 4–5 years, which is too late considering that the brain is most susceptive to interventions during the first two years of life. Currently, diagnosis of NDDs is based on observed behaviors and symptoms, but identification of objective biomarkers would allow for earlier screening. In this longitudinal study, we investigated the relationship between repetition and change detection responses measured using an EEG oddball task during the first year of life and at two years of age, and cognitive abilities and adaptive functioning during preschool years (4 years old). Identification of early biomarkers is challenging given that there is a lot of variability in developmental courses among young infants. Therefore, the second aim of this study is to assess whether brain growth is a factor of interindividual variability that influences repetition and change detection responses. To obtain variability in brain growth beyond the normative range, infants with macrocephaly were included in our sample. Thus, 43 normocephalic children and 20 macrocephalic children were tested. Cognitive abilities at preschool age were assessed with the *WPPSI-IV* and adaptive functioning was measured with the *ABAS-II*. Time–frequency analyses were conducted on the EEG data. Results indicated that repetition and change detection responses in the first year of life predict adaptive functioning at 4 years of age, independently of head circumference. Moreover, our findings suggested that brain growth explains variability in neural responses mostly in the first years of life, so that macrocephalic children did not display repetition suppression responses, while normocephalic children did. This longitudinal study demonstrates that the first year of life is an important period for the early screening of children at risk of developing NDDs.

## Introduction

Infancy is a crucial period in brain development since the brain is growing fastest and undergoes multiple changes during the first two years of life^1^. For the brain to develop healthily, dynamic and adaptive interactions between gene expression and environmental input are essential^2^. In this period, the brain is particularly vulnerable to environmental insults or pathogenic genetic alterations that can disrupt developmental processes. These disruptions can have long-lasting or permanent effects on brain structure and function, thus leading to neurodevelopmental disorders (NDDs)^1^. NDDs are defined as a heterogeneous group of disorders with onset in the developmental period, i.e. before school age, and include, among others, attention-deficit/hyperactivity disorder (ADHD), autism spectrum disorder (ASD), specific learning disorder (SLD), and language disorder (LD)^3^. Diagnosis of NDDs is based on observed behaviors and symptoms that are specified in either the Statistical Manual of Mental Disorders (DSM) or the World Health Organization (WHO) International Classification of Disease (CIM). For most of NDDs, children do not receive their diagnosis until 4–5 years of age^3^, which is late considering that the brain is the most susceptible to interventions during the first two years of life^4^. The WHO stated that the identification of infants at risk for neurodevelopmental disorders is a crucial starting point to provide early intervention^5^ and thus minimize motor, cognitive and emotional impairments in young children^4^. There is thus a need to establish objective biological markers for NDDS that would allow for earlier diagnosis. A biomarker, according to the basic definition elaborated by the BEST (*Biomarkers, EndpointS*, *and other Tools*) Resource, is a defined characteristic that is measured as an indicator of normal biological processes, pathogenic processes or responses to an exposure or intervention^6^. However, in the scope of this article, it would more appropriate to consider the definition of diagnostic biomarkers, which is defined as a biomarker that detects or confirms the presence of a disease or condition of interest, or identifies an individual with a subtype of the disease^6^. Identification of early biomarkers is challenging given that there is a lot of variability in developmental courses among young infants, this is why biomarkers validity and reliability for early prediction of neurodevelopmental disorders during infancy must be evaluated by criteria such as its relation to typical development and its sensitivity to developmental changes^7^.

Electroencephalography (EEG) offers several advantages compares to other neuroimaging techniques, such as its high temporal resolution, its usability with a wide range of population and age groups, and its low operating costs^8^. When it comes to identifying biomarkers, this neuroimaging technique has enabled researchers to establish typical patterns of brain development, then allowing the study of atypical brain development. Many parameters can be derived from EEG signal, whether it is task-locked or spontaneous recordings, such as absolute and relative spectral power, phase coherence, event-related potentials, entropy, cross-frequency coupling, and connectivity^9,10^. In the past years, researchers have invested efforts in finding EEG biomarkers for different NDDs. For example, in a review conducted by Wang et al.^11^, it is suggested that children with ASD show a U-shaped profile of electrophysiological power alterations, with excessive power at low-frequency (delta, theta) and high-frequency (beta, gamma) bands. Power in the middle-range frequency band (alpha) is, on the other hand, reduced. The authors attributed this U-shaped profile partially to the abnormal functioning of gamma-aminobutyric acid (GABA)ergic receptors in inhibitory circuitry. In a longitudinal study, the same team investigated the developmental course of resting EEG in infants at high-risk and low-risk for ASD. Using spectral power analyses, they demonstrated that at 6 months of age, spectral power was lower across all frequency bands (delta, theta, low alpha, high alpha, beta, and gamma) in high-risk infants. Moreover, the subsequent rate of change in spectral power in all frequency bands seemed to differ between the two groups, indicating that trajectories of change in EEG power may be a more robust biomarker^12^. More recently, researchers have found that intertrial coherence in the theta range during visual processing was reduced in infants who had a sibling diagnosed with ASD and who eventually received a diagnosis of ASD themselves. These results suggest that in the first year of life, reduced intertrial coherence in brain activity during face processing is associated with emerging autism^13^. Regarding children with ADHD or SLD, studies have found an increased theta/beta ratio^14,15^, which indicates altered top-down control of attention and affect^16,17^. SLD was also associated with an increased theta/alpha ratio, suggesting a lag in EEG maturation. Altogether, these results point toward a general slowing in EEG oscillations in children with SLD^14^.

One of the pathological neural processes identified in NDDs^18^ is the repetition response. This response is considered an experience-dependent neural change, reflecting Hebbian synaptic plasticity, considered to be the synaptic mechanism of learning^19,20^. With each presentation of the stimulus, the disparity between the ascending sensory input (bottom-up input) and the experience-dependent top-down prediction decreases resulting in more efficient information processing by the primary areas of the brain^19,21^. The change detection response refers to a larger neural response elicited when a repeated stimulus is followed by an unexpected stimulus (deviant stimulus). Repetition and change detection responses have been observed in young typically developing infants^22–24^ and they were found to be altered in various clinical populations such as patients with language impairment, ASD and X fragile syndrome^25–29^. Multiple cerebral factors could affect brain activity in these populations. In the last decade, little attention has been allocated to the frequent abnormal brain growth observed in these populations^30,31^. Macrocephaly is a relatively common clinical condition (5% of pediatric population^32^) defined as an abnormally large head with a circumference greater than 2 standard deviations above the mean for a given age and sex^33^. In most cases, this condition is familial and benign (i.e., idiopathic macrocephaly), and occurs in the absence of other neurological dysfunctions^34–36^. Results from past research have suggested impairments in the neurodevelopment of children with idiopathic macrocephaly leading to speech and language delay^37^, motor problems and neurodevelopmental dysfunction^38^, as well as arithmetic problems and visuomotor difficulty^39^. Whether brain overgrowth as an isolated trait affect brain activity remains to be studied.

In a recent study published by López‐Arango et al.^40^, the authors demonstrated modulation of repetition and change detection responses by adaptive abilities during infancy. However, it is still unknown if these neural responses could be associated with cognitive development later in childhood and whether these neural responses could be potential biomarkers for the early identification of children at risk of NDDs. The first aim of this study was to determine the relationship between repetition and change detection responses measured during the first year of life and at two years of age, and cognitive abilities and adaptive functioning during preschool years (4 years old) in normocephalic and macrocephalic infants. We hypothesized that infants who showed greater repetition responses (i.e., greater reduction of brain response between each presentation of the stimulus) and greater change detection responses (i.e., greater neural response to a deviant stimulus) during the two first years of life would have higher scores on the cognitive and adaptive functioning assessment tools 4 years of age.

The second aim of this study was to assess whether brain growth is a factor of interindividual variability that influences repetition and change detection responses, given the possible impacts of macrocephaly on the integrity of brain function, that could lead to sensory processing peculiarities, which the neural responses to repetition and change detection are the foundation. We hypothesized that children with increased brain growth during the first year of life would show less repetition suppression and change detection responses during the two first years of life, compared to normocephalic children.

## Materials

### Participants and procedure

We recruited 43 normocephalic children (24 males) and 20 macrocephalic children (11 males) aged three to eleven months to participate in this longitudinal study (see Table 1 for demographics). Normocephalic children were recruited at CHU Sainte-Justine’s birth unit, in daycares, and through social networks, while macrocephalic children were recruited at the medical imaging department, following a referral from their pediatrician. Developmental information was gathered from an in-house developmental questionnaire completed by the parents. All children were born at term with no pregnancy or delivery complications and had no significant health problems or suspicions of developmental delay. Parents gave informed written consent for themselves and for their infants prior to the study. The study was approved by the ethics, administrative and scientific committees of the Ste-Justine’s University Hospital Research Center and all experiments were performed in accordance with relevant guidelines and regulations.

**Table 1:** Demographics.

	Age at the first visit (mean (SD))	Age at the second visit (mean (SD))	Age at the third visit (mean (SD))
Control group			
Boys	5.4 (1.4)	23.7 (0.5)	48.3 (1.2)
Girls	4.9 (1.6)	23.9 (0.5)	48.1 (0.9)
Macrocephalic group			
Boys	6.7 (3.3)	24.1 (1.1)	48.6 (1.8)
Girls	6.4 (2.4)	24.2 (0.8)	48.1 (0.3)

This project is a longitudinal study including three visits to CHU Sainte-Justine at the *Neuroscience of Early Development (NED)* laboratory. The first visit took place during the infant’s first year of life (between 3–11 months), the second visit at two years of age, and the last visit is at four years of age. The oddball task administered at the first and second visits was analyzed to identify an EEG marker detectable before preschool age that would allow for earlier screening of children. An assessment of intellectual and adaptive abilities was done at the third visit.

### Intellectual and adaptive functioning assessment

To assess intellectual abilities of the children at 4 years of age, the *Wechsler Preschool and Primary Scale of Intelligence—Fourth Edition* (*WPPSI-IV*)^41^ was used, allowing us to obtain a Full-Scale IQ (*FSIQ*) score for each participant. Data for three participants is missing since they turned 4 years old during the COVID-19 confinement in 2020 so it was not possible to do the assessment. Adaptive functioning was assessed using the *Adaptive Behavior Assessment System—Second Edition* (*ABAS-II*)^42^, specifically the Parent/Primary Caregiver Form. The General Adaptive Composite (*GAC*) score was obtained for each participant. To avoid missing data during the COVID-19 confinement in 2020, this questionnaire was sent by mail to the families.

### EEG oddball task

**Figure 1:**
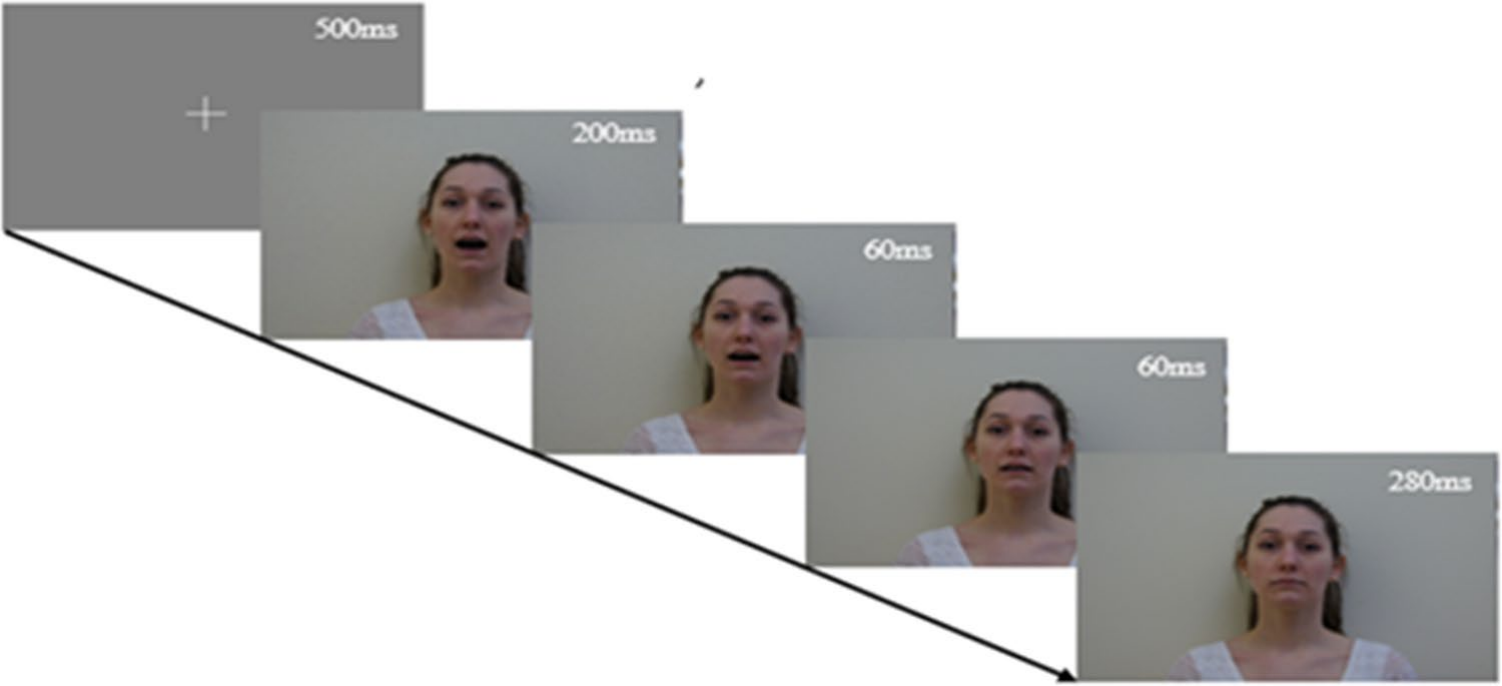
Experimental design. The task consisted of a man or a woman (pictured) articulating the vowel /a/. On each trial, infants were presented with three consecutive /a/ while the fourth vowel could be either /a/ or /i/. The sound lasted 200 ms, in synchrony with the first frame (mouth opened). Following the end of the sound, two frames of a mouth gradually closing were presented (60 ms). Finally, during the last 280 ms, a face with a closed mouth was presented. *Informed consent to publish identifying images was obtained.

The task consisted of audio-visual stimuli featuring a woman and a man alternating in the articulation of the vowels /a/ or /i/^24^. The audio-visual design served to maximize children’s attention. Stimuli were generated by a Dell Optiplex 790 PC using E-Prime 2.0 software (Psychology Software Tools Inc., Pittsburgh, PA, USA) on a 17″ screen placed at a viewing distance of 60 cm. A team member was present in the room with the infant to assure the child was looking sufficiently at the screen and that the child seemed attentive. Eye-tracking was monitored, indicating looking behavior to the experimenter outside the shielded room. The sounds were delivered at a sound pressure level of 70 dB through two speakers located laterally at 30 cm distance from the children’s ears. Infants were seated on their parent’s laps, in front of the screen. The onset of the auditory vowel coincided with a visual clip lasting 200 ms of the mouth fully opened. Following the end of the sound, two frames of 60 ms showing the mouth gradually closing were presented. During the next 280 ms, the face with a closed mouth was presented followed by the onset of the next vowel (Fig. 1). The task followed a local/global paradigm and consisted of 96 trials, 80 trials followed an *aaaI* pattern, which corresponds to a global standard with a local deviant (standard trial) and 16 trials followed an *aaaa* pattern, corresponding to a global deviant with a local standard. The order of the trials was pseudo-randomized and fixed across participants, making sure no *aaaa* trials would follow each other. To assess repetition and change detection responses, only standard trials that did not directly follow an *aaaa* trial were analyzed. See previously published articles from the same author for the original description of this method^43,44^.

### EEG recordings

EEG recordings took place in a dark soundproof experimental chamber. EEG was recorded continuously with a high-density EEG system containing 128 electrodes (Magstim EGI, Eugene, OR, USA). Altogether, the EEG net set-up and the recording took in average 60 min. EEG Signals were acquired and processed by a G4 Macintosh computer using NetStation EEG Software (Version 4.5.4). Data were acquired at a 1000 Hz sampling rate and an analog bandpass filter of 0.1–4000 Hz was applied. Impedances were kept below 40 kΩ^45^. Off-line signal processing and analyses were performed using MATLAB (Mathworks, Inc., Natick, MA) and the EEGLAB toolbox^46^. Data were digitally filtered with a lower-bound 0.5 Hz and a higher-bound 150 Hz and a 60 Hz notch filter. Twenty-eight electrodes containing muscular artifacts placed around the neck and face were excluded. Electrodes with a total standard deviation higher than 200 μV and lower than 2 μV were automatically removed. Channels with sporadic behavior were then manually removed during subsequent visual inspection. Data were re-referenced to the average reference. Eye movement and cardiac artifacts were rejected using semi-automatic independent component analysis (ICA) as implemented in the EEGLAB toolbox^46^. ICAs were calculated automatically, and rejection of the components was done manually. The data was segmented and cleaned in 800 ms time windows (− 200 to 600 ms), representing the time window of each stimulus with a short pre-stimulus segment. Epochs with voltage exceeding ± 200 μV were marked and a visual inspection of the segmented data (− 200 to 600 ms) was performed next to manually reject epochs with significant artefacts. Following epoch exclusion, an average of 79% epochs were kept for analyses. The data of 15 participants (visit 1: 11 participants, visit 2: 4 participants) were excluded from the analysis due to excessive artifacts or unusable recordings. See previously published articles from the same author for the original description of this method^43,44^.

### Regions of interest

Region of interest (ROI) was determined by using a PCA^47^ which identifies the most relevant and contributing cluster of electrodes while avoiding redundant dimensions^48^. PCA was used given the rapid maturation of the brain during childhood, which causes many functional changes, and the presence in our study of a clinical group for which almost no EEG literature exists. The analysis was carried out using a Varimax Rotation on IBM SPSS, Version 27 (IBM, Armonk, NY, USA). The observations consisted of the three presentations of the /a/ and the last /i/ in all participants for both testing time points, while the 99 channels were the dependent variables. The spatial PCA yielded 1 main factor, which explained 58% of data variance and included a cluster of 11 electrodes in the central region (E7, E30, E31, E37, E54, E55, E80, E87, E105, E106, E112). Moreover, the five electrodes explaining the most variability (E7, E31, E55, E80, E106) correspond exactly to the five electrodes that constitute the central region reported in the literature. Therefore, time–frequency analyses were conducted on this ROI since central region is known to be related to auditory processing^49^ and its coherent with past developmental studies showing that increased responses in central slow frequency are associated with learning and attention control in early childhood^50,51^. See previously published article from the same author for the original description of this method^43^.

### Time–frequency analyses

Time–frequency (TF) analyses were used as they provide temporal information on the activity of specific frequency bands. To avoid overlap between segments, a padding technique was implemented, in which the first spectral power value of each segment was added for the 800 ms period before and the last spectral power value for the 800 ms period after the segment, thus increasing its length to 2400 ms. Complex Gaussian Morlet’s wavelets transformation^52^ was computed to analyze the signal in the time and frequency domain. This specific wavelet convolution can be expressed as follows:

$$ M(t,\, f) = W * S = \int_{t} {W\left( {\frac{t - a}{b},\, f} \right)S(t) \cdot dt} $$

*M*(*t*, *f*) is a matrix of complex values (vectors) for a given time (t) and frequency (f). *S* was the signal as a function of time (t) and *W* corresponds to Morlet’s wavelet which is a complex exponential (Fourier) with a Gaussian envelope that undergoes a series of translations (a) and dilations (b) dependently on the frequency (f). The event-related spectral perturbation (ERSP) computation uses the complex values (amplitude and phase) given by Morlet’s wavelet transform as shown in the following formula calculating the power spectrum for each time and frequency point: $P(t,\;f) = 10\;\log_{10} (|M(t,\;f)|^{2} )..P(t,\;f)$ denotes TF power in terms of decibels (dB). Final TF maps were computed as follows: $TF = \frac{1}{N}\sum\nolimits_{n = 1}^{N} {P(t,\;f)}$. ERSP maps show mean log deviations from baseline power, averaged across participants for each testing time point. Investigated frequencies ranged from 3 to 55 Hz. The baseline was defined as the average of the segment [− 100: 500] ms for each epoch. Baseline correction was achieved by computing separately the mean power of each stimulus presentation (/a1/-/a2/-/a3/-/I/). This mean was subtracted from each time and frequency point to show variations in EEG activity.

Intertrial coherence (ITC), analogous to phase-locking value (PLV), allows the assessment of the strength of phase coherence or synchronization across trials in temporal and spectral domains^53^. The ITC computation uses only the phase of the complex values given by Morlet’s wavelet transform. To extract phase-locking information, ITC was computed according to Lachaux and his colleagues’ procedure^54^. ITC measures phase coupling across trials at all latencies and frequencies and is defined by: $ITC = \frac{1}{N}\left| {\sum\nolimits_{n = 1}^{N} {exp\;(j\theta \;(f,\;t,\;n))} } \right|$ where θ represents the phase for a given frequency (f), time point (t), and trial (n). The obtained values are always defined between 0 and 1. Phase-locking values close to 1 indicate strong inter-trial phase-locking, thus representing evoked activity while scores closer to 0 indicate a high inter-trial phase variability^54,55^. See previously published articles from the same author for the original description of this method^43,44^.

### Topographies

Past developmental literature showed that low frequencies are predominant in infants and that frequency boundaries are lower than in adults and shift with brain maturation. In fact, in infants and children, theta band varies between 3 and 5–6 Hz, alpha band varies between 6–9–10 Hz, and beta band includes frequency from 9 to 30 Hz^50,51,56–60^. On the other hand, few to no literature exists regarding exact time intervals of brain activity. The results found here and in our previous publications^40,43,61,62^ show theta and alpha activity after a post-stimulus delay, and beta activity early after stimuli onset. Thus, for selecting time intervals, ITC maps were created for each stimulus and testing time point (visit 1 and visit 2) to assess the time–frequency windows (TFW) in which EEG activity showed greater variation in synchronization across trials and testing time points. Based on these previous studies on infants EEG oscillations and the ITC maps, the 3 following TFWs were selected since they captured most of the activity: theta (3–5 Hz, 100–400 ms), alpha (5–10 Hz, 100–400 ms), and beta (10–30 Hz, 0–200 ms). We used ITC maps since it takes evoked responses into consideration, which means brain activity that is phase-locked to the stimulus onset. For each TFW of interest, the time–frequency spectrogram for each electrode available for each subject, and each stimulus were calculated. The power of each electrode was then averaged across all the participants for each TFW. An averaged topography including all the subjects was then computed for each stimulus (/a1/-/a2/-/a3/-/I/) based on the mean power of each TFW, for both ERSPs and ITCs. Topographic map inspection confirmed that the central region showed large brain activity. See previously published articles from the same author for the original description of this method^43,44^.

### Statistical analyses

#### Descriptive statistics and preliminary analyses

Descriptive statistics and preliminary analyses were performed using SPSS Statistics, version 27 (IBM Corp., Armonk, NY, USA). Preliminary analyses were conducted to assure there were no differences between groups regarding annual family income (*t*(57) = 1.4, *p* = 0.17, *d* = 0.41). Differences between boys and girls regarding age at the three testing time points, for each group were also assessed (normocephalic group : visit 1: *t*(41) = − 1.1, *p* = 0.27, *d* = − 0.34, visit 2: *t*(41) = 1.1, *p* = 0.28, *d* = 0.33, visit 3: *t*(39) = − 0.52, *p* = 0.61, *d* = − 0.16, macrocephalic group : visit 1 : *t*(18) = − 0.21, *p* = 0.83, *d* = − 0.10, visit 2: *t*(18) = 0.09, *p* = 0.93, *d* = 0.04, visit 3: *t*(17) = − 0.78, *p* = 0.44, *d* = − 0.37). Since age the first visit varies between three months and eleven months and that neural responses change significantly during infancy du to brain maturation, this variable should be controlled in the main analysis. Moreover, when statistically assessing difference between groups regarding age at the first visit, results show a near significant difference with a medium effect size based on Cohen’s D (*t*(23,9) = − 1.3, *p* = 0.058, *d* = − 0.67). Although there were 3 missing participants for the FISQ and 15 missing participants for the EEG oddball task, no imputation was conducted since it has been shown that it is not necessary to use multiple imputations to handle missing data before applying mixed model analysis on longitudinal data^63^.

#### Linear mixed models

The main analyses were performed using SPSS Statistics, version 27 (IBM Corp., Armonk, NY, USA). Linear mixed models (LMM) analyses were chosen because they allow for unequal numbers of measurements, and the analysis uses all observations that are available for a given participant. Moreover, LMM allows the measuring time points to vary for different participants^64,65^. LMM analyses were conducted on ERSP values since they showed greater variability compared to ITC values.

To verify our first hypothesis, four general models were performed: Visit 1—*FISQ*; Visit 1—*GAC*; Visit 2—*FISQ*; Visit 2—*GAC*. In each model, the general pattern of responses across the standard trial (/a1/-/a2/-/a3/-/I/) was assessed and the association between the pattern of responses and intellectual abilities and adaptive functioning at preschool age was verified. Stimulus presentations, TFW, groups, and interactions between these variables were the fixed effects (4 presentations × 3 TFW × 2 groups), whereas FSIQ and GAC scores were the dependent variables. Age at the first visit (only for Visit 1 models) and sex were introduced sequentially as predictors to the models and a chi-square likelihood ratio was used to verify model fit improvement.

To assess our second hypothesis, two LMMs, one for each testing time point (visit 1 and visit 2), were conducted to assess the difference in the general pattern of responses across the standard trial (/a1/-/a2/-/a3/-/I/) between normocephalic and macrocephalic children. The models included stimulus presentations, TFW, and the group as the fixed effects (4 presentations × 3 TFW × 2 groups) and so were the interactions between these effects.

For all LMMs, intercept and slope were allowed to vary randomly. Restricted maximum likelihood (REML) was used since it tends to result in unbiased estimates of the variances and covariances^66^. The first-order autoregressive covariance structure (AR1) provided the best model fit for the Visit 1—*FISQ* and Visit 1—*GAC* models, whereas the heterogeneous first-order autoregressive covariance structure (ARH1) provided the best model fit for the Visit 2—*FISQ* and Visit 2—*GAC* models.

#### ANOVA

In relation to our second hypothesis, ANOVAs were conducted separately for each TFW, with each stimulus as the dependent variable and groups as the group variable to determine which stimulus (/a1/-/a2/-/a3/-/I/) varies the most between the two groups.

## Results

### Brain responses, cognitive abilities, and adaptive functioning

#### Linear mixed models

##### Visit 1 (3–10 months)—FISQ

The best model fit was found by including age at the first visit and sex as predictors [χ^2^ (3, N = 52) = 17.8, p < 0.001]. The results indicated that there was no significant relationship between the response to the entire standard trial (/a1/-/a2/-/a3/-/I/) measured at the first testing time point and *FISQ* scores measured at 4 years of age (F (1, 556.97) = 2.4, *p* = 0.12). No effect of age at the first testing time point, sex, groups or TFW were found.

##### Visit 1 (3–10 months)—GAC

The best model fit was found by including age at the first visit and sex as predictors [χ^2^ (3, N = 52) = 12.5, p < 0.01]. A significant interaction was found between the response to the entire standard trial (/a1/-/a2/-/a3/-/I/) measured at the first testing time point and *GAC* scores measured at 4 years of age (F (1, 575.9) = 6,90, *p* < 0.009), suggesting that infants with higher *GAC* scores showed a U-shaped pattern in response to the entire standard trial (/a1/-/a2/-/a3/-/I/). Infants with lower *GAC* scores tended to show a steady repetition suppression response between each presentation of the four stimuli, and therefore they showed no change detection response. No effect of age at the first testing time point, sex, groups or TFW were found.

##### Visit 2 (24 months)—FISQ

The best model fit was found by including sex as a predictor [χ^2^ (3, N = 59) = 8.9, p < 0.05]. The results indicated that there was no significant relationship between the response to the entire standard trial (/a1/-/a2/-/a3/-/I/) measured at the second testing time point and *FISQ* scores measured at 4 years of age (F (1, 523.89) = 2.96, *p* = 0.086). No effect of sex, groups nor TFW were found.

##### Visit 2 (24 months)—GAC

The best model fit was found by including sex as a predictor [χ^2^ (3, N = 59) = 18.1, p < 0.001]. No significant relationship between the response to the entire standard trial (/a1/-/a2/-/a3/-/I/) measured at the second testing time point and *GAC* scores measured at 4 years of age (F (1, 678) = 2.25, *p* = 0.13) was found. No effect of sex, groups nor TFW were found.

### Brain responses and brain growth

#### Linear mixed models

##### Visit 1 (3–10 months)

The best model fit was found by including age at the first visit and sex as predictors [χ^2^ (3, N = 52) = 17.3, p < 0.001]. The results suggested that there was a significant difference between groups regarding response to the entire standard trial (/a1/-/a2/-/a3/-/I/) measured at the first testing time point (F (1, 598.8) = 8.47, *p* = 0.004). Responses of the normocephalic infants followed a more U-shaped pattern compared to macrocephalic infants who showed a more linear and steady response. No effect of age at the first testing time point, sex, or TFW were found.

##### Visit 2 (24 months)

The best model fit was found by including sex as a predictor [χ^2^ (3, N = 59) = 16.7, p < 0.001]. No significant difference between groups regarding response to the entire standard trial (/a1/-/a2/-/a3/-/I/) measured at the second testing time point (F (1, 693) = 1.80, *p* = 0.180) was found. This result suggests that macrocephalic children showed a pattern of response similar to normocephalic children when tested at two years of age.

#### ANOVA

##### Visit 1 (3–10 months)

**Figure 2:**
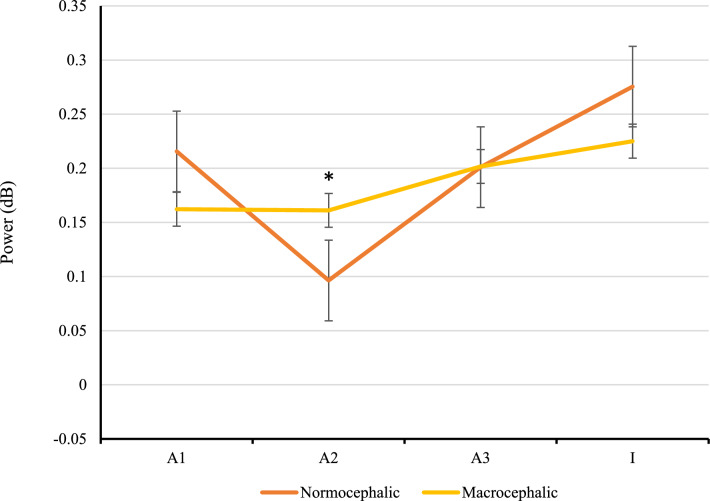
Averaged power (ERSP) of the theta TFW (3–5 Hz, 100–400 ms) for each group, at the first visit, in the central region. Normocephalic infants (orange line) showed a U-shaped pattern in response to the entire standard trial (/a1/-/a2/-/a3/-/I/) while macrocephalic infants (yellow line) showed a steady response between the first and the second presentation of the /a/. Error bars represent standard error. *p < 0.01.

**Figure 3:**
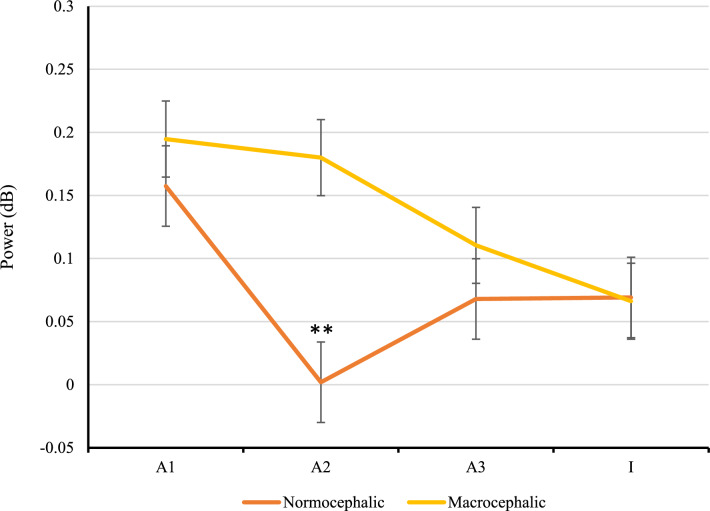
Averaged power (ERSP) of the beta TFW (10–30 Hz, 0–200 ms) for each group, at the first visit, in the central region. Normocephalic infants (orange line) showed a U-shaped pattern in response to the entire standard trial (/a1/-/a2/-/a3/-/I/) while macrocephalic infants (yellow line) showed a relatively steady response between the first and the second presentation of the /a/. Error bars represent standard error. **p < 0.001.

The results of the one-way analysis of variance indicated that there was a significant difference between groups regarding the response to the /a2/ in the theta frequency band (F (1, 109) = 7.73, *p* = 0.006) and in the beta frequency band (F (1, 50) = 13.35, *p* < 0.001). Normocephalic infants showed significantly less power on the second presentation of the stimulus /a/ compared to macrocephalic infants, who tended to show no repetition suppression between the first and the second presentation of the /a/ (Figs. 2 and 3).

##### Visit 2 (24 months)

**Figure 4:**
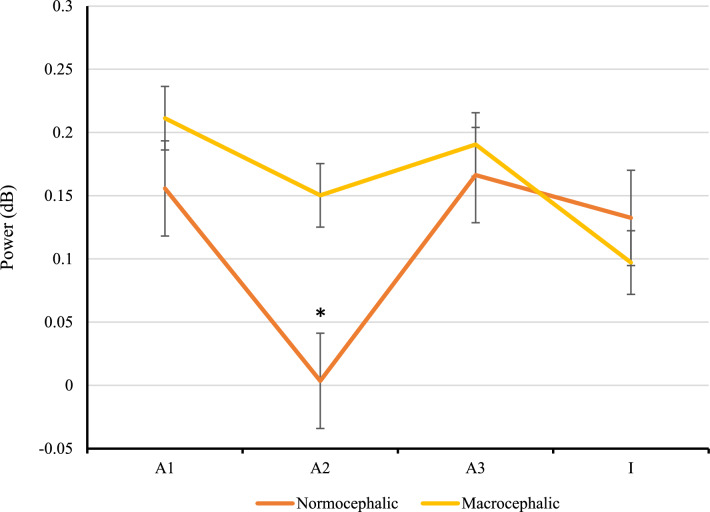
Averaged power (ERSP) of the alpha TFW (5–10 Hz, 100–400 ms) for each group, at the second visit, in the central region. Both normocephalic children (orange line) and macrocephalic children (yellow line) showed a U-shaped pattern in response to the entire standard trial (/a1/-/a2/-/a3/-/I/). Macrocephalic children displayed a less steep repetition suppression response between the first and the second presentation of the stimulus. Error bars represent standard error. *p < 0.01.

A significant difference between groups regarding the response to the /a2/ (F (1, 57) = 9.08, *p* = 0.004) in the alpha frequency band was found in the one-way analysis of variance. Again, normocephalic children showed significantly less power on the second presentation of the stimulus /a/ compared to macrocephalic children, who showed a less steep repetition suppression response between the first and the second presentation of the stimulus compared to the control group (Fig. 4). See supplementary material for the figures of the 3 TFW for each group at each testing time point.

## Discussion

This study aimed to investigate the relationship between repetition and change detection responses measured during infancy, and cognitive abilities and adaptive functioning in childhood. Our results indicated that when measured during the first year of life, repetition and change detection responses were not associated with cognitive abilities but predicted adaptive functioning at preschool age. Infants who displayed the expected U-shaped pattern in response to the entire standard trial (/a1/-/a2/-/a3/-/I/)^43^ were the ones who had the highest *GAC* scores (better adaptive functioning) at 4 years of age. On the other hand, no change detection response when presented with the /I/ was elicited in infants who had the lowest *GAC* scores. These results indicate that deviant responses should be analyzed in conjunction with the standard responses in neurodevelopmental studies. These findings are further in line with recent literature demonstrating that in children and adolescents with ASD, reduced neural responses to changes in stimuli input were associated with deficits in adaptive functioning^67^. Past studies have also suggested that in various clinical populations repetition and change detection responses were altered^25–29,68–70^ and that children with NDDs (ADHD, autism, patients with tics or language disorders) have significantly lower adaptive functioning scores compared to children without NDDs^71^. Based on past results and results of this study, it is possible to hypothesize that the capacity to flexibly adapt to life circumstances and the surrounding environment may be underlain by basic brain functions such as repetition and change detection responses. Moreover, the association found in our study was found regardless of the groups (normocephalic or macrocephalic), meaning that repetition and change detection responses could help identify children at risk of impaired adaptive functioning both in the general and clinical infant population. Regarding the absence of associations between repetition and change detection responses and cognitive abilities, one possible explanation could be that repetition suppression response is systematic in children with IQ scores within the normal range. In a study conducted by Orekhova et al.^72^, they demonstrated that children with autism in the normal IQ range showed repetition suppression between the first and the second presentation of auditory clicks while the ones with low IQ scores (within the limit and extremely low range) did not show repetition suppression. Moreover, studies comparing control groups with low IQ clinical groups found repetition suppression in the control group (IQ scores within the normal range) but no or altered repetition suppression response in clinical groups with low IQ scores^25,27,28^. Our results are thus in line with the literature since, for the most part, children in our sample displayed repetition suppression in response to the entire standard trial (/a1/-/a2/-/a3/-/I/) in the first year of life and most of the children had FISQ scores within the normal range at 4 years of age.

The second aim of this study was to assess whether brain growth is a factor contributing interindividual variability to repetition and change detection responses since it is developmental courses among young infants are well known to display a lot of variability. Our findings suggested that in the first year of life, macrocephalic infants differ from normocephalic infants in the way they respond to the entire standard trial (/a1/-/a2/-/a3/-/I/). Indeed, normocephalic infants showed the expected U-shape pattern but macrocephalic infants showed no repetition suppression or repetition enhancement between the first and the second presentation of the stimulus. A possible explanation could be that macrocephalic infants, compared to normocephalic infants, did not draw enough information from the first /a/ so they were not able to form a strong representation of the stimulus, thus explaining why they showed steady responses or repetition enhancement on the second presentation of the /a/. Indeed, it has been demonstrated that the direction of the repetition effect depends on the amount of information that can be drawn from the first presentation of the stimulus in order to establish a strong representation. If the representation needs to be strengthened, there is no repetition suppression, but rather repetition enhancement^73,74^. At the second visit, the results then suggested a normalized response in the macrocephalic children, responding in a U-shaped pattern as the normocephalic children. Nevertheless, a significant difference was found between the groups in response to the second presentation of the /a/, indicating that the U-shaped pattern was steeper in normocephalic children. In sum, the rate of brain growth was a factor of interindividual variability that influences repetition responses only during the first year of life but not anymore at two years of age. Moreover, our results also suggested that repetition and change detection responses at the second visit were not associated with cognitive abilities nor with adaptive functioning at preschool age, thus indicating that these neural responses lose their predictive power between the first and second year of life. These findings are in line with the ones of Knickmeyer et al.^1^ indicating that the first year of life represents a critical period for brain development since brain volume increases the most during this period. Based on their results, the authors suggested that there was an urgent need to identify children at risk for neurodevelopmental disorders within the first year of life to maximize the effects of interventions.

In this study, we were able to demonstrate that repetition and change detection responses have the potential to help identify children at risk for neurodevelopmental disorders in the first year of life. Our paradigm is sensitive to abnormal brain growth and can be linked to adaptive development in preschool years. Therefore, these cerebral responses meet the early prediction criteria of a diagnosis biomarker. Overall, we showed that these brain responses relate to typical development, they are sensitive to developmental change, they relate to a specific condition which may reflect an underlying neural mechanism. Nevertheless, more clinical populations should be evaluated with the task to quantify reliability, sensitivity, and specificity.

As future perspectives, since the effectiveness of a biomarker relies on its ability to stratify individuals into various diagnostic categories, in the future, the ability of the neuronal response to the entire standard trial to classify individuals according to their symptoms, level of functioning, cognitive impairment, or diagnosis should be established. Moreover, it would be relevant to relate the electrophysiological response to other measures beyond intellectual functioning and adaptive skills. This could, for example, include more specific cognitive functions such as executive functions, attention and memory, or questionnaires to measure autistic symptoms, ADHD symptoms, behavioral problems, or aspects of temperament.

Even though adding sex as a predictor improved the model fit significantly for all the models tested, no sex effect or interaction was found. This result is in line with a recent study showing no effect of sex on repetition suppression responses in infants under 12 months of age^44^. However, Clarke et al.^75^ have found differences in EEG maturation between boys and girls. The total power, in delta, theta, and alpha frequency bands, increased with age in boys but decreased in girls, suggesting a faster rate of change in girls^76^, possibly explaining why sex as a predictor fitted our data better but did not have an effect on repetition and change detection responses.

This study has some strengths as well as some limitations. We used a longitudinal design which allowed us to assess the development of the same individuals from the first months of life to 4 years of age, making it is possible to think that our findings highlight maturational effects. One limitation is the unequal group size, caused by the challenges of recruiting macrocephalic children and missing data, which can lead to unequal variance between groups. However, the linear mixed model analyses used in this study allowed to minimize the impact of unequal group size and missing data since it can deal easily with unbalanced longitudinal data sets^66,77^. Also, given the relatively small sample size, it limits the generalizability of the results. Finally, it is possible that ROIs and TFWs selection caused a circularity problem since data was first analyzed to select a subset, and then the same subset was reanalyzed to obtain the results^78^. However, ROIs and TFWs were selected both by data-driven approach and based on the literature.

## Conclusion

Taken together, these findings demonstrated that only the repetition and change detection responses in the first year of life are predictive of adaptive functioning at 4 years of age. Further research should focus on testing this relationship in populations with various neurodevelopmental disorders to establish the sturdiness of these neural responses as biomarkers. Moreover, differences between macrocephalic and normocephalic infants in response to the oddball task have been observed only during the first year of life. When tested at two years of age, macrocephalic children responded in a very similar way as normocephalic children, suggesting that brain growth influences the neural response mostly in the early months of life.

## Date availability

The datasets generated during and/or analysed during the current study are available from the corresponding author on reasonable request.

## Supplementary Information

Supplementary Information.
