# Supplementary material for: EEG repetition and change detection responses in infancy predict adaptive functioning in preschool age: a longitudinal study
Source: Sci Rep. 2023 Jun 20;13:9980. doi: 10.1038/s41598-023-34669-9 (PMC10282122; doi:10.1038/s41598-023-34669-9)
Supplement: Supplementary file 1 — Supplementary Information. [file 41598_2023_34669_MOESM1_ESM.docx]

**Title:** EEG repetition and change detection responses in infancy predict adaptive functioning in preschool age: A longitudinal study.

**Author names and affiliations: ***Florence Deguire^1,2,3^, Gabriela Lopez-Arango^1,2,3^, Inga Sophia Knoth^3^, Valérie Côté^1,2,3^, Kristian Agbogba^3,4^, Sarah Lippé^1,2,3^

^1^ Psychology department, University of Montreal, Marie Victorin Building, 90 Vincent-D’Indy Avenue, Montreal, Quebec, Canada

^2^ Pôle en neuropsychologie et neuroscience cognitive et computationnelle (CerebrUM), University of Montreal, Marie Victorin Building, 90 Vincent-D’Indy Avenue, Montreal, Quebec, Canada

^3^ Research Center of the CHU Sainte-Justine, University of Montreal, 3175 Chemin de la Côte-Sainte-Catherine, Montreal, Quebec, Canada

^4^ École de technologie supérieure, University of Quebec, 1100 Notre-Dame W, Montreal, Quebec, Canada

**Supplementary methods and results section**

**Methods**

**Regions of interest**

Each spatial factor represents a specific spatial configuration of brain activity and the factor loading corresponds to the spatial factor’s contribution to the original variables (i.e. how much the spatial factor accounts for the voltage recorded at each electrode). These spatial configurations are defined by considering electrodes with the highest loading factors ^1^. A group of electrodes was identified as a region of interest (ROI) when the loadings of these electrodes were superior to 0.7, corresponding to more than 50% of the data variance being explained. The spatial PCA yielded 11 factors. The first factor explained 58% of data variance and included a cluster of 11 electrodes in the central region (see Fig 1).


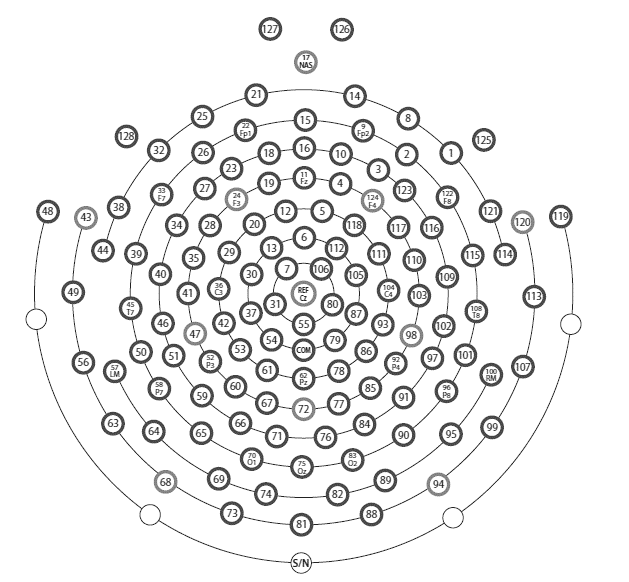


**Figure 1. PCA results.** Factor 1 (black dots) included 11 electrodes in the central region and explained 58% of the data variance.

**Time-frequency analyses**

**
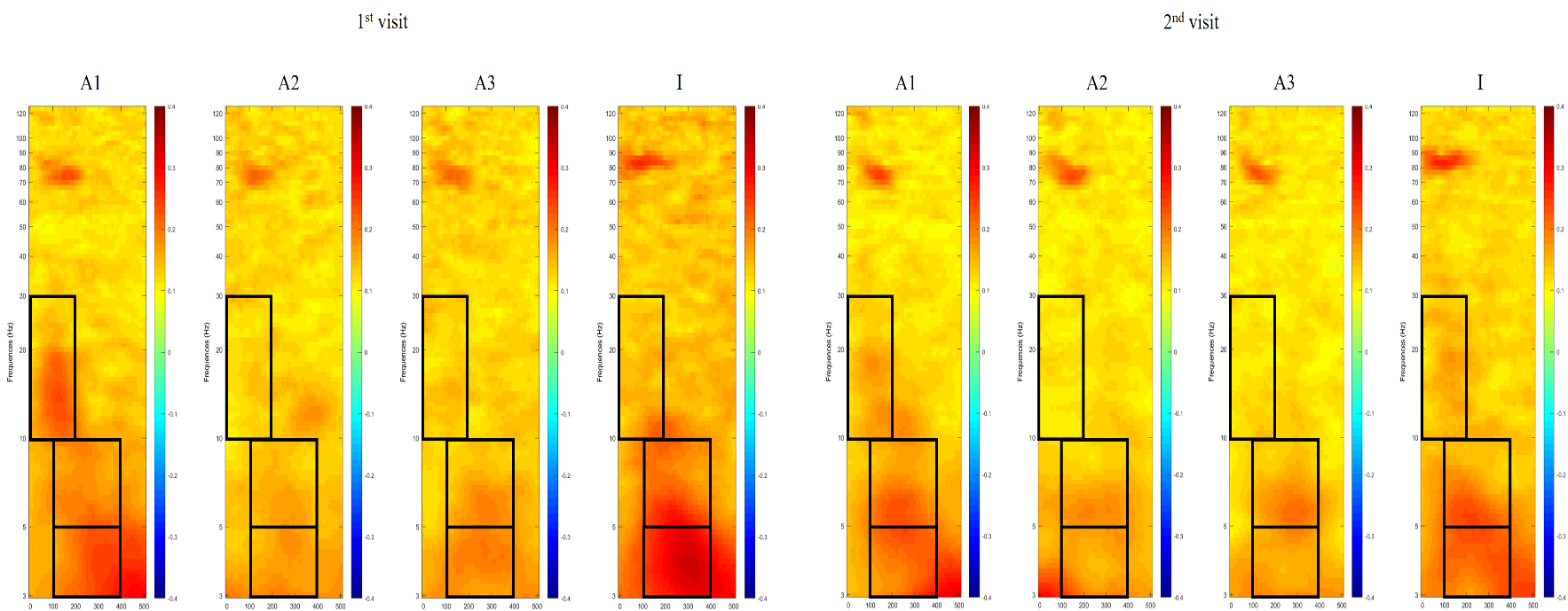
**

**Figure 2. ITC maps showing selected time-frequency windows for each testing time point, for the normocephalic group.** Averaged synchronization of the first visit (between 3 to 10 months old) and the second visit (24 months old) for each stimulus presentation. Rectangles illustrate the selected time-frequency windows: theta (3-5 Hz, 100-400ms), alpha (5-10Hz, 100-400ms), and beta (10-30Hz, 0-200ms).

**
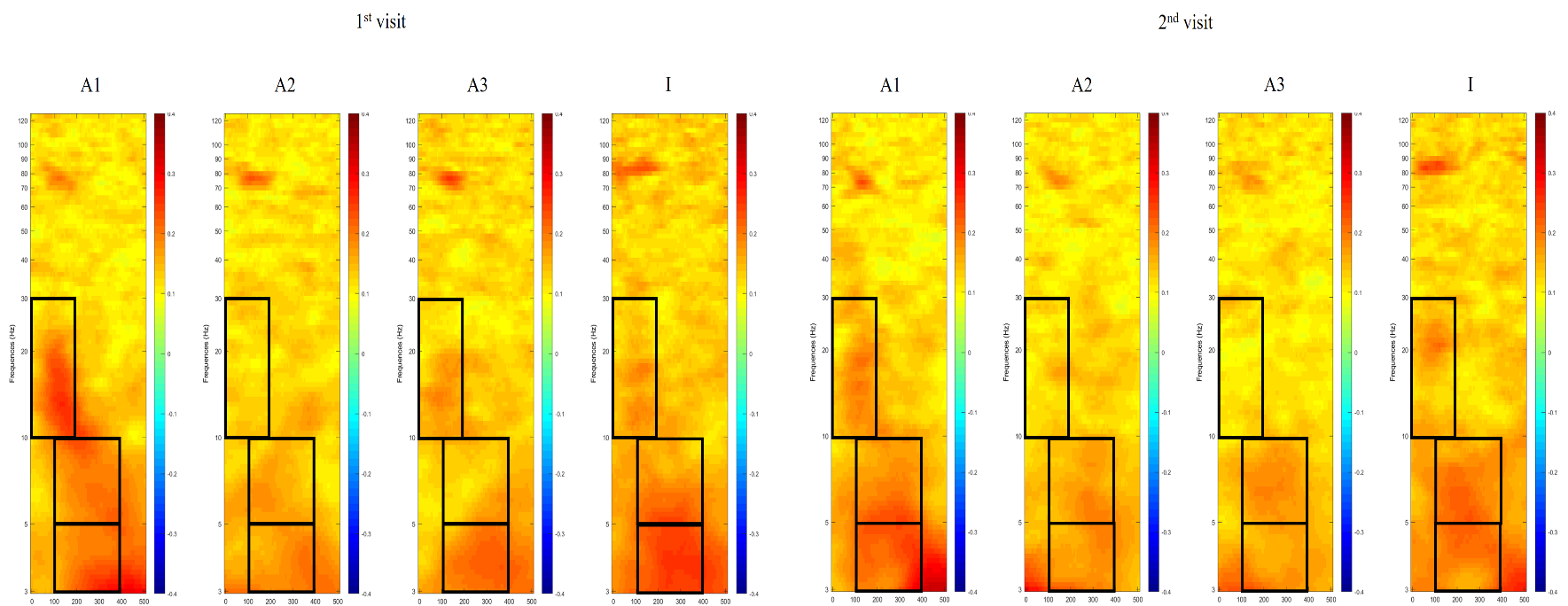
**

**Figure 3. ITC maps showing selected time-frequency windows for each testing time point, for the macrocephalic group.** Averaged synchronization of the first visit (between 3 to 10 months old) and the second visit (24 months old) for each stimulus presentation. Rectangles illustrate the selected time-frequency windows: theta (3-5 Hz, 100-400ms), alpha (5-10Hz, 100-400ms), and beta (10-30Hz, 0-200ms).

**Topographies**

**
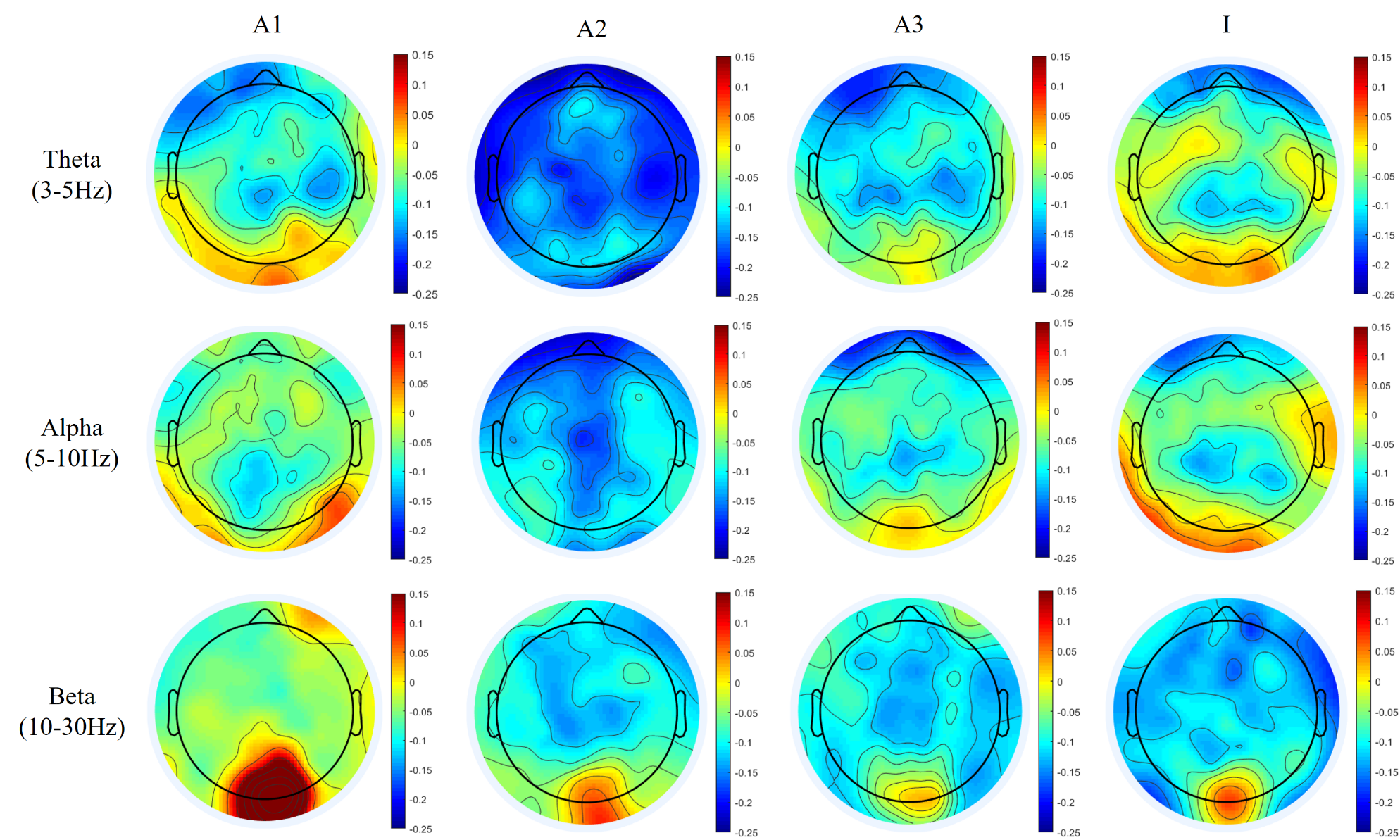
**

**Figure 4. ERSP topographies of the normocephalic group.** Averaged event-related spectral perturbation (ERSP) topographies across testing time points for each stimulus presentation and each TFW.


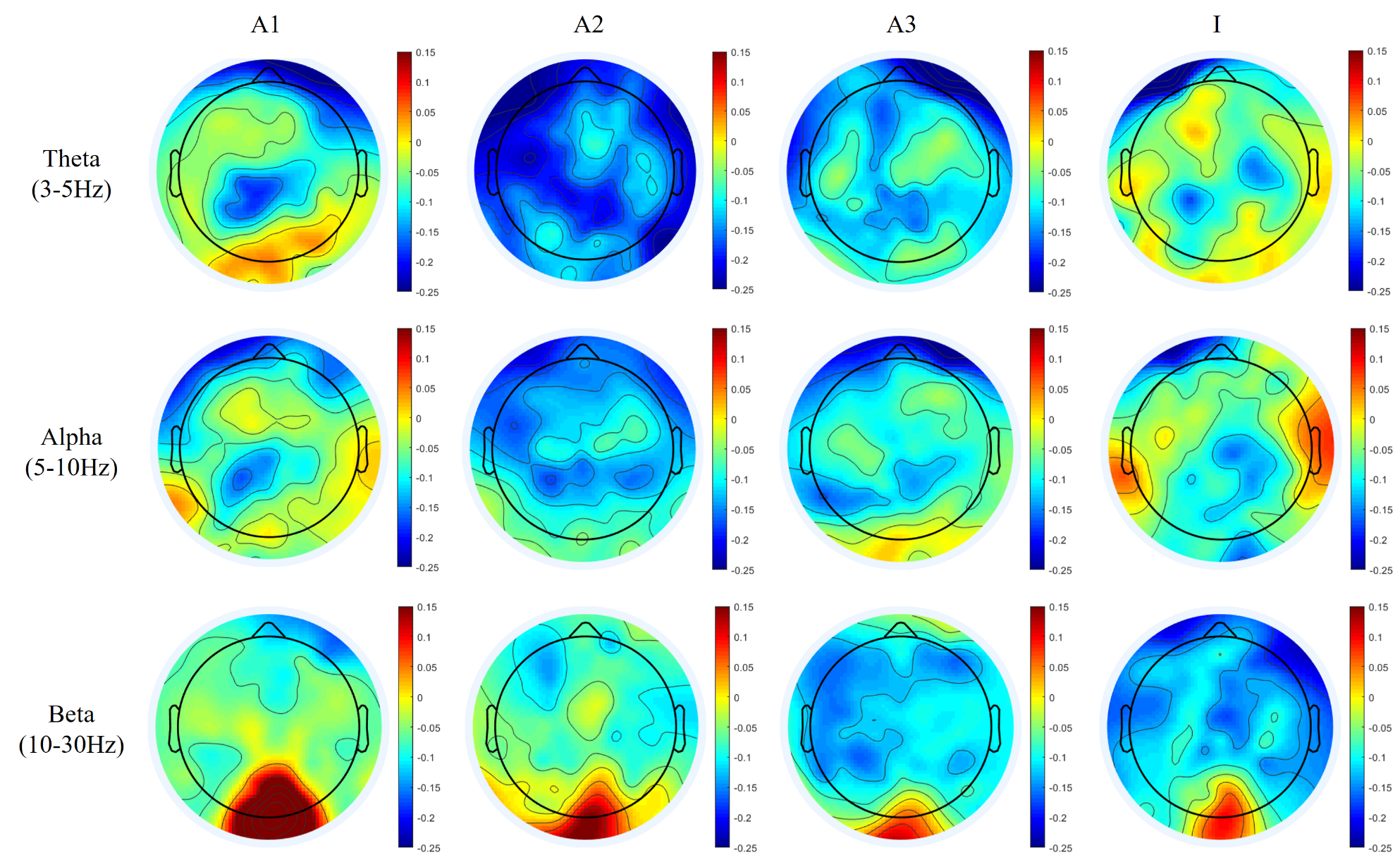


**Figure 5. ERSP topographies of the macrocephalic group.** Averaged event-related spectral perturbation (ERSP) topographies across testing time points for each stimulus presentation and each TFW.

**
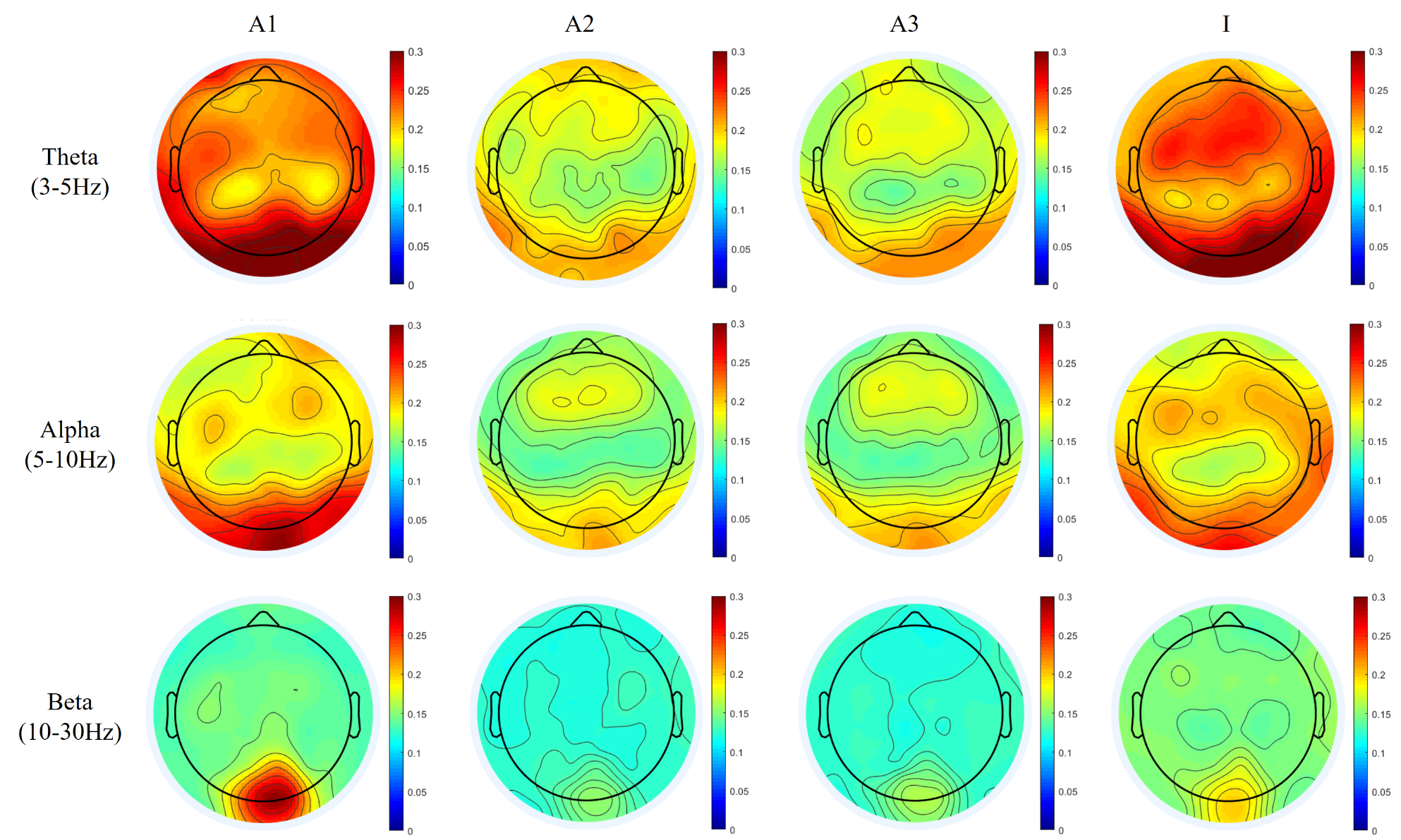
**

**Figure 6.** **ITC topographies of the normocephalic group.** Averaged synchronization (ITC) topographies across testing time points for each stimulus presentation and each TFW.


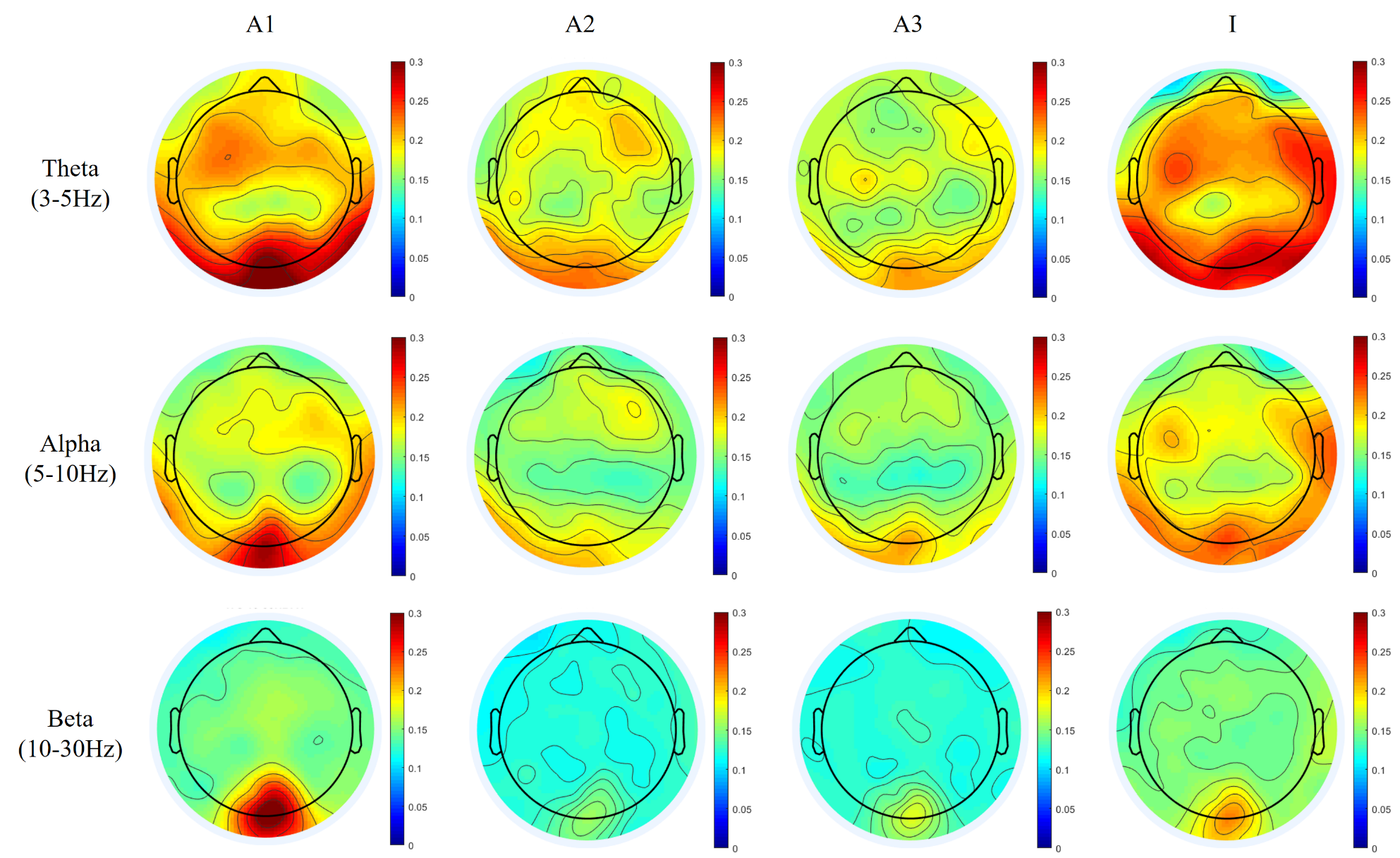


**Figure 7.** **ITC topographies of the macrocephalic group.** Averaged synchronization (ITC) topographies across testing time points for each stimulus presentation and each TFW.

**Results**


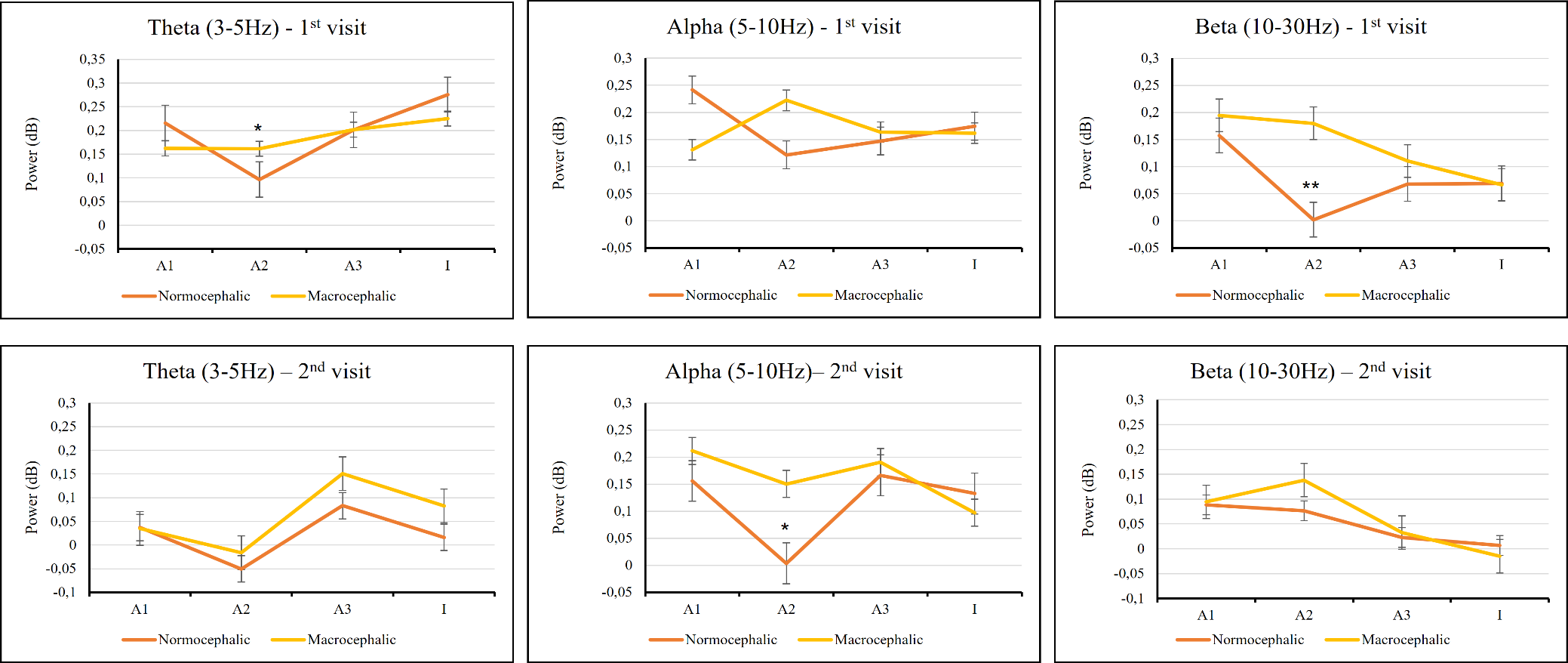
**ANOVA**

**Figure 8.** **Averaged power (ERSP) for each TFW and each group, for the two testing time points, in the central region.** In the first year of life, normocephalic infants showed the expected U-shape pattern but macrocephalic infants showed no repetition suppression or repetition enhancement between the first and the second presentation of the stimulus. At two years of age, the results then suggested a normalized response in the macrocephalic children, responding in a pattern similar as the one of normocephalic children.
